# The new preparation method for paraffin-embedded samples applying scanning electron microscopy revealed characteristic features in asthma-induced mice

**DOI:** 10.1038/s41598-022-12666-8

**Published:** 2022-05-31

**Authors:** Ken Wakai, Kazuhiko Azuma, Chiaki Iwamura, Maihulan Maimaiti, Kosuke Mikami, Kei Yoneda, Shinichi Sakamoto, Sanae Ikehara, Takashi Yamaguchi, Kiyoshi Hirahara, Tomohiko Ichikawa, Toshinori Nakayama, Yuzuru Ikehara

**Affiliations:** 1 Department of Pathology, Graduate School of Medicine, Chiba University, Chiba, 260-8670 Japan; 2 Department of Urology, Graduate School of Medicine, Chiba University, Chiba, 260-8670 Japan; 3 Department of Immunology, Graduate School of Medicine, Chiba University, Chiba, 260-8670 Japan; 4 Cellular and Molecular Biotechnology Research Institute, National Institute of Advanced Industrial Science and Technology, Tsukuba, 305-8565 Japan; 5 AMED-CREST, AMED, 1-8-1 Inohana, Chuo-ku, Chiba, 260-8670 Japan

**Keywords:** Electron microscopy, Cell signalling

## Abstract

In bronchial asthma patients, mucous cell metaplasia (MCM) and fibrosis occur in the bronchial epithelium and interstitium, respectively. The mucus and collagen fibers are identified by Periodic acid-Schiff stain (PAS) or Sirius red stain on optical microscopy. On a scanning electron microscope (SEM) observation, formalin-fixed-paraffin-embedded specimens have high insulation, thereby attenuating the scattered electron signals leading to insufficient contrast. Moreover, there were no staining methods for SEM observation, which characterizes the changes in epithelium and interstitium by enhancing the scattered electrons. In this study, we established a method of coating osmium thin film on pathological tissue specimens using plasma chemical vapor deposition technology. This method ensured the intensity of scattered electron signals and enabled SEM observation. Furthermore, we found that morphological changes in MCM and interstitial fibrosis could be characterized by Grocott stain, which we optimized to evaluate pathological remodeling in bronchial asthma. Using these techniques, we compared asthma-induced mice with Amphiregulin (Areg) knockout mice, and found that Areg induce MCM, but the production of Grocott-stain-positive substrate in the interstitium is Areg-independent. The method developed in this study provides an understanding of the pathological spatial information linked to the ultrastructural changes in cells and interstitium due to disease-related signaling abnormalities.

## Introduction

Most pathological specimens fixed with formalin and embedded in paraffin are observed with an optical microscope. However, the insulating nature of pathological specimens makes scanning electron microscope (SEM) observation difficult because accelerated electrons charge the specimen surface, causing a charge-up phenomenon that distorts the image. Therefore, SEM observation of highly insulating samples including semiconductors and organic materials, requires pretreatment, such as highly conductive coating applied to the sample to release the charge on the sample surface^1^. Pretreatment is done by vapor deposition or sputtering^2^, but due to the complex three-dimensional structure of pathological specimens' and cells' surfaces on glass slides, a uniformly thin film coating is difficult. This is solved by using ionic liquids^3^, which are increasingly used for SEM observation because of their ability to penetrate the pathological specimen, impart conductivity, and resist evaporation even in a vacuum. Ionic liquid, because they are in liquid form, has the advantage of familiarity with entire specimens with complex surface structures such as pathological specimens, and does not require special equipment for processing. However, it has the problem of obtaining a solid signal intensity due to its low reflection coefficient. On the other hand, Nanosuit^4^ in liquid form can solve ionic liquids' low reflection coefficient^5^, while it is difficult to uniformly coat the tissue surface with thickness from 50 to 200 nm, resulting in lost SEM signals from the cell microstructure.

In tissue observation using SEM, it is necessary to optimize the coating that does not mask the tissue image while preventing charge-up. However, mucous cell metaplasia (MCM) and fibrosis in asthma are characterized by increased production of acidic glycans, and the sample is easily charged. Since it is difficult to form a uniformly thin film by vapor deposition or sputtering, it has been difficult to understand the spread of MCM and fibrosis from the central to the peripheral bronchi ultra-micromorphologically. The pathology of asthma with a background of chronic inflammation is assessed by Periodic acid-Schiff (PAS) staining of mucus produced from MCM, in which bronchial epithelial tissue is replaced by glandular cells^6^, but PAS-positive mucus vesicles could not be identified with the resolution of optical microscopy. Furthermore, analysis by transmission electron microscope (TEM) is limited to a very narrow space with a limited observation area^7^, whereas SEM can observe the spread from the central to the peripheral bronchi. However, the scattered electron signals from mucus vesicles and fibrosis are very low, and SEM observation is difficult due to the lack of staining methods which visualize the epithelium and stroma changes by enhancing the scattered electrons.

In this study, to detect and analyze the spread of MCM and fibrosis in asthma using SEM, we established the pretreat on osmium (Os) plasma chemical vapor deposition (pCVD) technique of the tissue specimens and optimized it to form a conductive thin uniform film. Moreover, for bronchial gland mucus and fibrous tissues with a low reflection coefficient, we established a method of increasing the reflected electrons' signal intensity by reducing silver ions by introducing aldehyde groups to glycans through chromic acid oxidation. This staining method, commonly referred to as Grocott staining, is also called fungal staining^8,9^. We succeeded in performing pCVD under conditions in which the introduced silver ion into mucus and fibrosis enhanced the recognition of the ultra-micromorphologies. Furthermore, using the established pCVD method, we investigated the ultrastructural difference in the lung tissues between wild-type and Amphiregulin deficient mice (Areg KO) mice^10^ after the sensitization with house dust mite (HDM)^11^. Because Areg is a driving factor for allergic inflammation and fibrosis triggered by HDM sensitization, AregKO mice developed minimal inflammation, but fibrosis is barely prominent^12^.

## Methods

### Bronchial asthma model and AregKO model

We used bronchial asthma model mice sensitized with HDM (HDM-sensitized B6 mouse). Eight- to twelve-week-old C57BL/6 mice were given Dermatophagoides pteronyssinus extracts (Greer Laboratories) intranasaly: 200 μg on day 0 and 100 μg on days 7 and 14. 50 μg of HDM was then given twice per week for 4 weeks, as described previously^11^.

We also used mice in which Amphiregulin (Areg) was knocked out to suppress asthma development and sensitized by HDM (HDM sensitized AregKO mouse), as described by Morimoto et al.^12^. Dr. David C. Lee (University of Georgia, GA, USA) initially developed AregKO mice and kindly provided them to Chiba University^10^. The Animal Care and Use Committee of Chiba University approved all animal care. The experiments were performed based on the Fundamental Guidelines for Proper Conduct of Animal Experiments and Related Activities in Academic Research Institutions under the jurisdiction of the Ministry of Education, Culture, Sports, Science and Technology of Japan. All methods are reported in accordance with ARRIVE guidelines.

Paraffin block specimens of lung tissue excised from the above HDM-senstitized B6 and AregKO mice were provided and used from the department of Nakayama et al. Slides were prepared from paraffin-embedded blocks by slicing 2.5–2.75 µm thick sections at a level where bronchial epithelial cilia could be clearly observed.

### Immunohistochemistry

Histological analysis by hematoxylin and eosin (H&E) staining was performed based on the protocol of Ikehara and others^13^. For Grocott staining, reaction times of reagents appropriate for SEM observation were determined based on the protocol used for fungal staining. The reagents and kits used were Muto Chemical's Methenamine Silver Set No. 40662 (MUTO PURE CHEMICALS, Tokyo, Japan).

### Observation equipment and conditions

For histological analysis of HE-stained specimens, an optical microscope was used. For ultrastructural analysis, a low vacuum SEM (TM4000Plus) (Hitachi High-Tech Corporation, Tokyo, Japan) was used with accelerating voltages of 5, 10, and 15kv. Glass slides for pathological diagnosis (PM) (Matsunami, Osaka, Japan) and glass sides fore SEM observation (EM) (Nissin EM, Tokyo, Japan) were selected. Neo Osmium Coater (NEOC) (Meiwafosis, Tokyo, Japan) was used to coat the specimens, and the thickness of the osmium coating was adjusted for observation. We compared and verified each condition shown in Table 1 to find the best condition for pathological specimen observation.

**Table 1:** Verified conditions for SEM observation.

1. Glass slides	PM (Matsunami)	EM (Nissin EM)
2. Os coating thichness	2.5 nm	5.0 nm
3. SEM acceleration voltage	5kv and 10kv	10kv and 15kv

## Results

### Optimal coating conditions and accelerating voltage for observing Grocott-stained specimens using SEM

Grocott staining is used to detect fungi by oxidizing the hydroxy group (-OH) of the fungal wall sugar chain with chromic acid to form an aldehyde group (-CHO), which then reacts with silver ions. Since Grocott staining is known to react with fungal walls, mucus, and amorphous interstitial substrates, we investigated the conditions of an Os coating suitable for SEM observation of Grocott-stained tissue specimens.

First, the staining intensity of mucus and fibrous tissue was weak under the same conditions as for fungal staining (Supplementary Fig. 1). To overcome this, we set the paraffin dissolver at 65 °C, heated the methenamine silver solution in advance (15 to 20 min), and fixed the reaction time of methenamine silver to 35 to 45 min. This operation prevented the non-specific reaction of silver particles, and at the same time, it enhanced the staining intensity of mucus and fibrous tissue.

The thickness of the Os coating was adjusted by the voltage and time of the plasma discharge in the NEOC osmium coater; a discharge of 9 Pa for 10 s produces a film thickness of 2.5 nm, and a discharge of 20 s produces a film thickness of 5.0 nm^14^.

For the glass slides on which the specimens were placed, we used EM, which contains indium, to improve conductivity and prevent the specimens from being charged during SEM observation. We used PM for paraffin pathology specimen observation.

**Figure 1:**
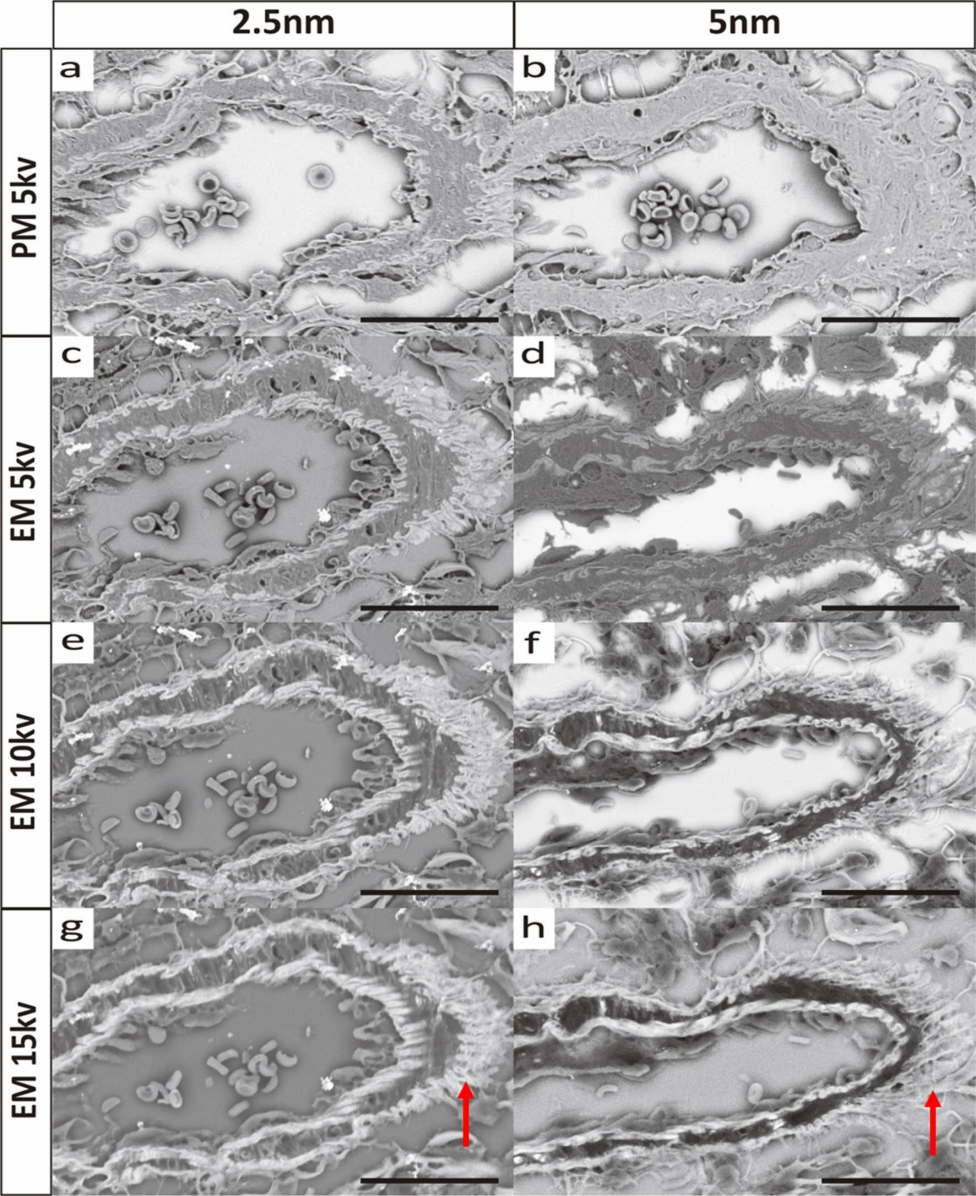
SEM images of Grocott-stained paraffin specimens of bronchial companion artery (Ar) resected from wild-type mice without any treatment. (**a**)–(**h**) indicates the same area. (**a**), (**b**) PM, 5kv, coating thickness 2.5 nm and 5.0 nm. (**c**), (**d**) EM, 5kv, coating 2.5 nm and 5.0 nm. (**e**), (**f**) EM, 10kv, coating 2.5 nm and 5.0 nm. (**g**), (**h**) EM, 15kv, coating 2.5 nm and 5.0 nm. (**a**, **b**) For PM, 5kv, 5.0 nm, image was uniform and difficult to observe the microstructure, while 2.5 nm provided contrast. (**a**, **c**) The same 5kv, 2.5 nm coating provided images with higher contrast in EM than in PM. (**b**, **d**) In both PM and EM, the 5kv, 5.0 nm coating provided images with higher contrast. In both PM and EM, 5kv, 5.0 nm coating did not depict microstructure. (**e**, **f**) EM, 10kv, gave clear images, but 2.5 nm gave better contrast. (**g**, **h**) EM, 15kv showed image disturbance due to charge-up (arrows). All scale bars show 25 μm length.

We searched for the most appropriate conditions for SEM observation of Grocott-stained pathological paraffin specimens based on the above. The conditions were compared for the companion artery of bronchopulmonary tissue resected from wild-type mice without any treatment (Fig. 1).

When aligned with an acceleration voltage of 5kv and a coating of 2.5 nm, we obtained images with higher contrast with EM than with PM, suggesting that EM is more suitable for SEM observation (Fig. 1a, c).

Using EM, images with stronger contrast were obtained at 2.5 nm compared to 5.0 nm in all voltages (Fig. 1c–f). With EM and 2.5 nm coating, microstructures were clearly observed at 10kv, but the image was distorted due to charge-up at 15kv. Thus, EM, 10kv, 2.5 nm coating was the best condition to observe bronchial pathology specimens (Fig. 1e, g).

### Observation of bronchial tissue in asthma model

In bronchial asthma tissue, chronic allergic inflammation leads to MCM of the bronchial epithelium, causing mucus production from the bronchial epithelium. However, there have been no reports of ultrastructural observations of this process. Therefore, to prove the existence of fibrosis and MCM in bronchial asthmatic lung tissue, we performed SEM observations using bronchial lung tissue from HDM-sensitized B6 and AregKO mice.

**Figure 2:**
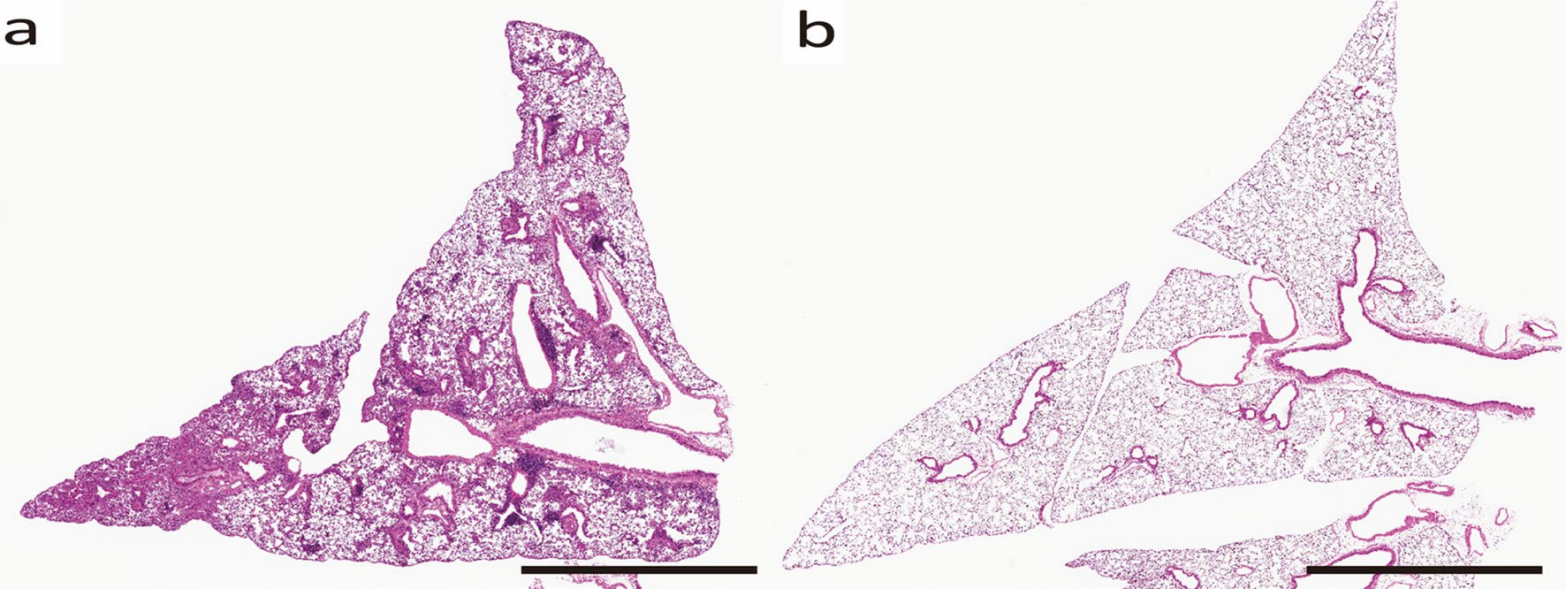
Optical microscopy images of bronchial tissue (HE staining) from HDM-sensitized B6 mice (**a**) and HDM-sensitized AregKO mice (**b**). (**a**) B6 mice show increased fibrosis. (**b**) AregKO mice show the suppressed fibrosis. All scale bars show 2 mm length.

Optical microscopy images of bronchial tissues of B6 and AregKO mice sensitized by HDM are shown (Fig. 2). In HDM-sensitized B6 mice, fibrosis is prominent. In contrast, in HDM-sensitized AregKO mice, fibrosis is barely observed because the Areg-Osteopontin pathway is blocked^12^^.^ To evaluate the ultrastructural changes in these specimens, they were stained with Grocott stain on EM and observed with a 2.5 nm Os coating at an acceleration voltage of 10 kV.

**Figure 3:**
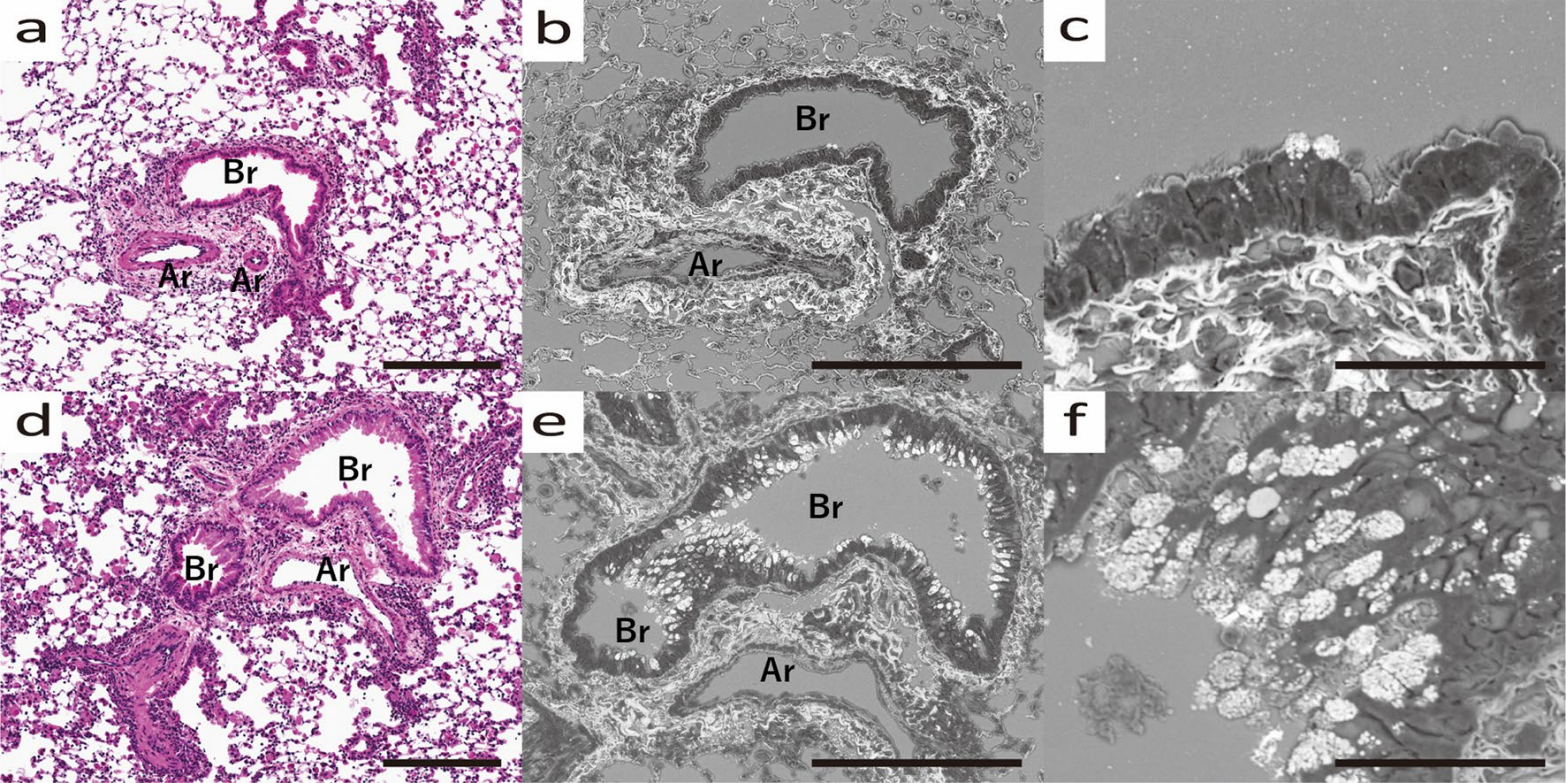
Morphological changes of pulmonary tissue in HDM-sensitized B6 mouse. (**a**) Optical microscope image of Bronchi (Br) and accompanying blood artery (Ar), HE stained. (**b**) SEM image of Br and Ar, Grocott stained. EM, 10kv, 2.5 nm. (**c**) Magnified SEM image of Br and Ar. (**d**) Optical microscope image of another Br and Ar, HE stained. (**e**) SEM image of Br and Ar, Grocott stained. f) Magnified image of Br and Ar. (**a**–**c**) HDM-sensitized B6 mouse developed fibrosis surrounding the entire bronchus, especially near the artery, and mucus production only from the same area. (**d**–**f**) Mucus vesicles are being produced from bronchial epithelial cells. All scale bars show 200 μm length.

In bronchial asthma lung tissue, the appearance of fibrosis around the bronchi is a characteristic feature (Fig. 3a), and the Grocott stain revealed a marked increase in fibrous tissue around the outer membrane of the blood vessels. In addition, fibrosis was observed around the entire vessel wall, while the peribronchial fibrosis tended to be stronger near the vessels and weaker elsewhere (Fig. 3b). Furthermore, mucous vesicles with strong Grocott stain, which could not be observed in HE, were observed, and their secretion from epithelial cells was imaged (Fig. 3d, e, f). Some bronchi showed mucus secretion only from the areas surrounded by significant fibrosis (Fig. 3c).

**Figure 4:**
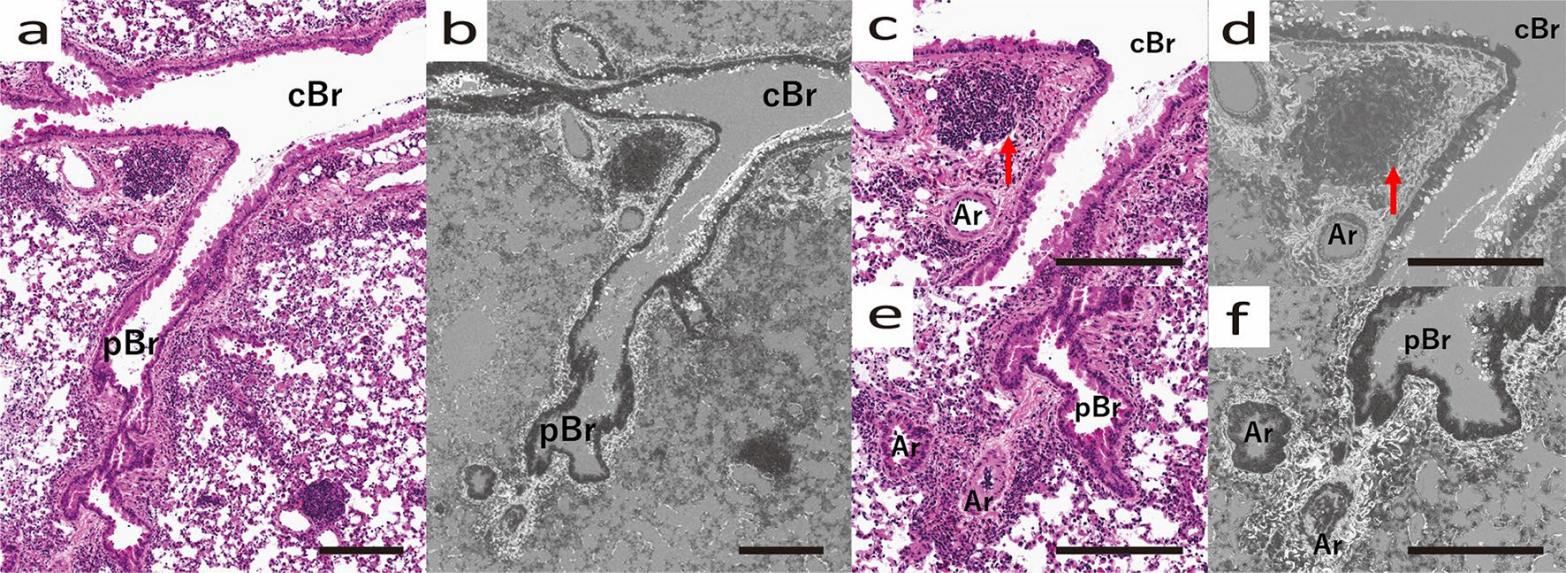
Morphological changes of central to peripheral bronchi in HDM-sensitized B6 mouse. (**a**) Optical microscope image (HE stained) and (**b**) SEM image (Grocott stained) of the bronchi. Central bronchus (cBr) and peripheral bronchus (pBr) are shown. (**c**) Optical microscope image (HE stained) and (**d**) SEM image (Grocott stained) of cBr and Ar, Lymph node (arrow) between cBr and Ar is seen. (**e**) Optical microscope image (HE stained) and (**f**) SEM image (Grocott stained) of pBR and Ar. (**a**, **b**) Mucus production was higher in the central part and lower in the peripheral part. (**c**, **d**) Lymph nodes are enlarged in the amorphous matrix between the extravascular lumen and bronchi. All scale bars show 200 μm length.

The fibrosis was extensive around the companion vessels, regardless of whether the bronchi were central or peripheral. However, mucus production was high on the central side of the bronchi and low on the peripheral side (Fig. 4a, b). In the fibrosis on the central side, lymph nodes were observed between the extravascular lumen and the bronchi (Fig. 4c, d). In the periphery of the lymph nodes, fibrosis was strong, and mucus production was abundant, but it was not particularly abundant just below the lymph nodes (Fig. 4e, f).

### Observation of bronchial tissue in AregKO model

**Figure 5:**
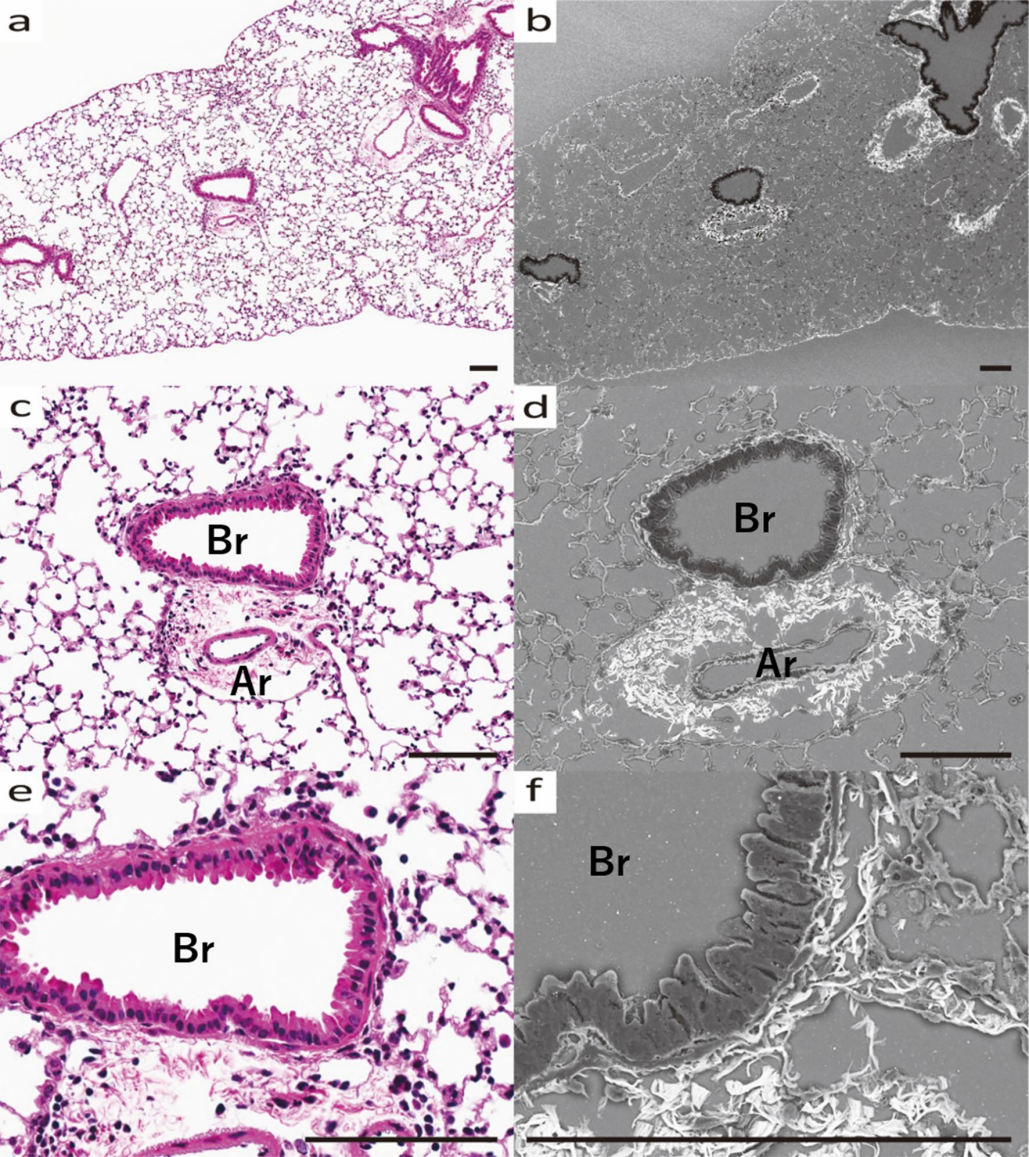
Bronchial tissue of HDM-sensitized AregKO mice. (**a**) Optical microscope image (HE stained) and b) SEM image (Grocott stained) of pulmonary tissue (**c**) optical microscope image (HE stained) and (**d**) SEM image (Grocott stained) of Br and Ar. (**e**) Higher magnified optical image (HE stained) and (**f**) SEM image (Grocott stained) of Br. (**a**, **b**) Weak peribronchial fibrosis throughout. (**c**–**f**) Little perivascular fibrosis and no spillover into the peribronchial area. Wide interstitial space. All scale bars show 100 μm length.

**Figure 6:**
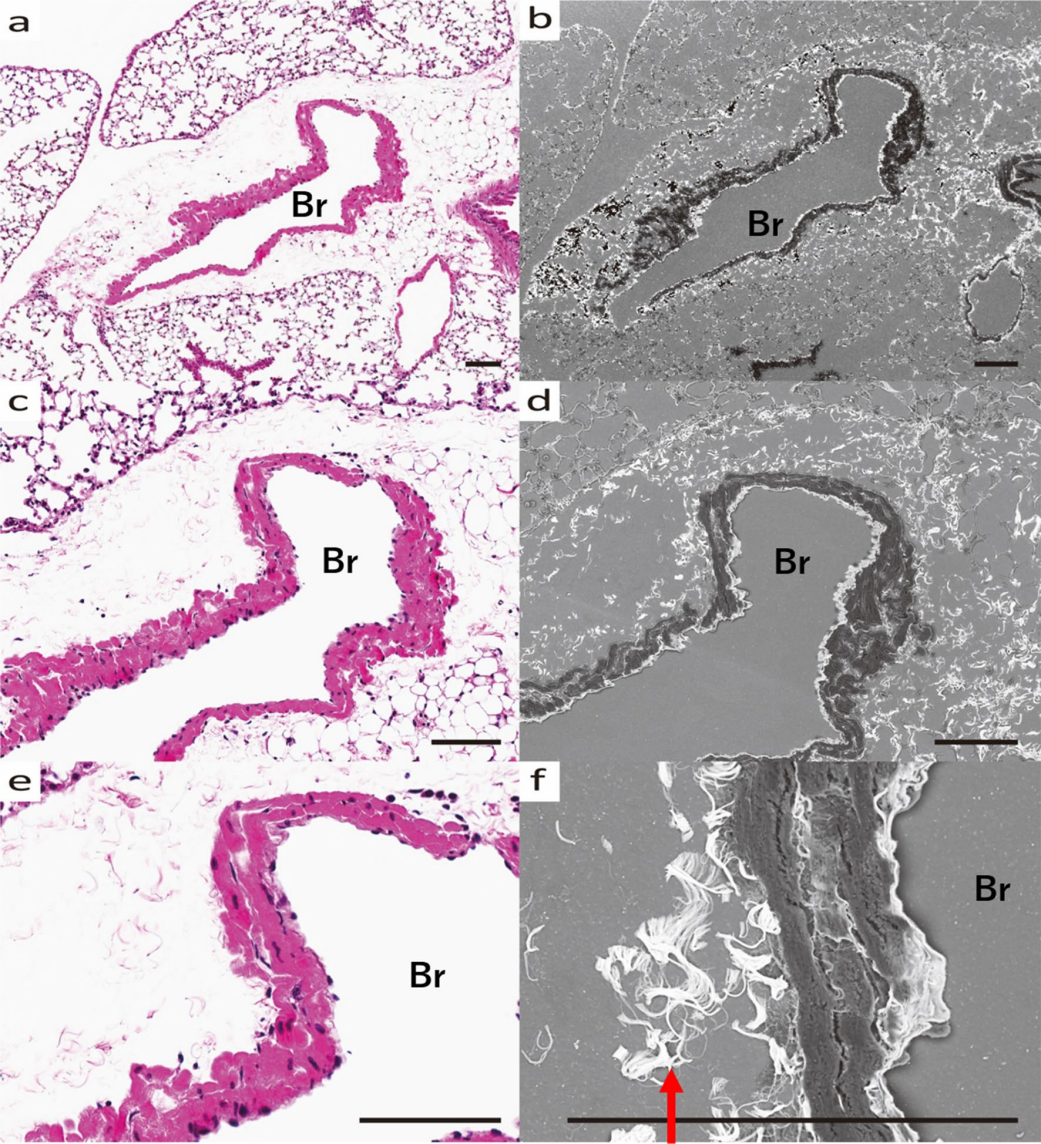
Bronchi (Br) and surrounding stroma of AregKO mice (HDM-sensitized). (**a**) Optical microscope image (HE stained) and (**b**) SEM image (Grocott stained) of pulmonary tissue. (**c**) Optical microscope image (HE stained) and (**d**) SEM image (Grocott stained) of Br. (**e**) Optical microscope image (HE stained) and (**f**) SEM image (Grocott stained) of enlarged Br and interstitium. (**a**–**d**) Edematous changes around the bronchi are seen. (**e**, **f**) Amorphous substrate of the interstitium (arrow) is stained without fibrous tissue. All scale bars show 100 μm length.

In HDM-sensitized AregKO mice, the fibrosis around the bronchial wall was generally weaker than in HDM-sensitized B6 mice, and the gap between fibers was also noticeable. (Fig. 5a, b, e, f) The fibrosis of the bronchial wall around the companion vessel was less and did not extend to the entire circumference of the bronchus (Fig. 5c, d). Indeed, HDM-sensitized AregKO mice did not show the increasement of fibrous tissue, but developed edematous changes in the interstitium around the bronchi, in contrast with HDM-sensitized B6 mice. Edematous changes in the interstitium did not contain collagen fibers but were filled by amorphous substrate that is positive for Grocott-stain (Fig. 6).

**Figure 7:**
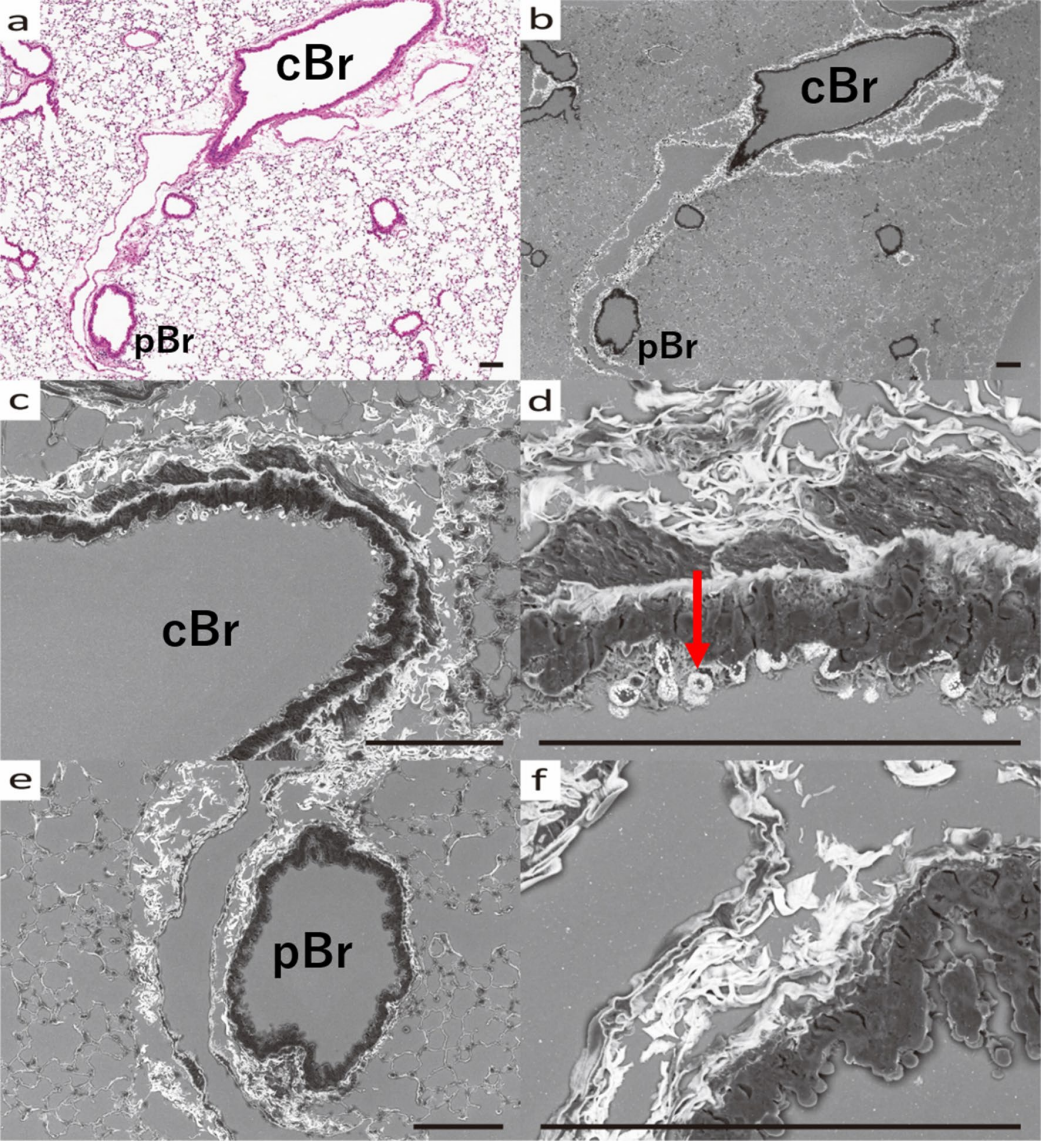
Morphological changes of the central to peripheral bronchi of AregKO mice (HDM-sensitized). (**a**) Optical microscope images (HE stained) and (**b**) SEM image (Grocott stained) of cBr pBr. (**c**) SEM image of cBr. (**d**) Higher magnified image of cBR. (**e**) SEM image of pBr. (**f**) Higher magnified image of pBr. (**a**, **b**) Peribronchial fibrosis is uniformly small from central to peripheral. (**c**, **d**) Small amount of mucus production (arrow) is observed in the central region, but it is much less than that in B6 mice. (**e**, **f**) As in B6 mice, mucus production in the peripheral region is smaller than that in the central region, and is almost absent. All scale bars show 100 μm length.

In a continuous view from central to peripheral, a small amount of mucus production was observed on the central side. However, almost no mucus production was observed on the peripheral side. Lymph node formation was also poor and unremarkable (Fig. 7).

## Discussion

In this study, we established a low-vacuum SEM observation using Grocott stain. This technique enables us to simultaneously observe ultramicroscopic morphology and identify mucus and fibrous tissue, which was previously impossible. Using this technique, we demonstrated mucus secretion from epithelial cells by MCM in the bronchial tissue of a bronchial asthma mouse model. By identifying the distribution of fibrosis, we found an increase in the amorphous matrix around the bronchial companion vessels, indicating that the perivascular stroma may be the primary site of fibrosis in bronchial asthma. This technique can be applied to observe any pathological paraffin tissue that can be sectioned and is expected to help elucidate the pathology involving mucus production and fibrosis.

The insulation of pathological specimens prepared from formalin-fixed, paraffin-embedded sections is high, and it has been difficult to obtain histological characteristics using SEM. However, we solved this problem by coating Grocott-stained tissue specimens with Os. In fact, by visualizing the Grocott stain-positive mucous vesicles and interstitial matrix by SEM, we understood the ultrastructural changes in cells and the interstitium linked to histological spatial information. While this is similar to what has been reported in earlier studies (ultramicro-morphological mucus detection by TEM)^7^, we have achieved a linkage to spatial histological information over the entire lung field that these previous studies could not achieve. In addition, previous studies in SEM have failed to confirm the formation of mucus vesicles that appear in asthma models. However, by confirming this, we have successfully linked morphological evidence to the proposed pathological remodeling in asthma.

In this study, we were able to coat Os on histopathological specimens. Although plasma coating of pathological specimens has been difficult due to their insulation, we have achieved a thin coating using the NEOC osmium coater even for complex structures such as thin sections on glass slides. Although there have been reports of Os-coated inorganic specimens with a resolution equivalent to that of TEM^15^, the application to pathological tissues has been insufficient. In this respect, the impact of this study is that it has made it possible to proceed with pathological analysis using SEM. The limitation of SEM observation of pathological tissues is that sputtering and vapor deposition methods can only be used to a limited extent because of the heat sensitivity of the samples. Ionic liquids and Nanosuit do not cause thermal damage to the specimen, allowing for observing pathological tissue specimens by SEM. However, ionic liquids are not sufficiently conductive, so the signal intensity is low, resulting in a darker and less clear image. We also examined Nanosuit and found that it was difficult to control the film thickness and to obtain the contrast of fibrous tissue; the charge-up was a problem at high magnification (Supplementary Fig. 2).

Os coating is a method of depositing Os ionized at low temperature by plasma, which does not cause thermal damage to the sample^16,17^. In contrast to the wet process of ionic liquids and Nanosuit, which results in micrometer-scale film thickness, the dry process allows the film thickness to be controlled at the nanometer level, which we believe solves the problem of superimposed microstructures. In fact, in the NEOC osmium coater, the film thickness is adjusted according to the time of plasma discharge. It has been confirmed that the greater the film thickness, the more conductive the film and the more effective it is to prevent charge-up. However, we have also found that greater film thickness requires higher acceleration voltage, which reduces image resolution. Therefore, we conclude that the optimal conditions for observing Grocott-stained specimens prepared from formalin-fixed, paraffin-embedded specimens are an Os film thickness of 2.5 nm and an acceleration voltage of 10 kV (Fig. 2).

SEM analysis of histopathological specimens allowed us to identify MCM and interstitial changes in an asthma mouse model, from which we conclude that the perivascular interstitium is primarily where changes occur in bronchial asthma. In fact, the increase in amorphous substrate positive for Grocott staining was strongest around the bronchial arteries (Fig. 3). In view of the previous literature, inflammatory cells migrating to the bronchial epithelium and extravasation due to inflammatory stimuli mainly in the stroma around the companion artery may contribute to these changes^18^ Notably, we found an increase in amorphous substrate positive to Grocott stain in bronchial asthma, which is not under the control of Areg signaling (Fig. 6). Furthermore, SEM analysis in this study confirmed that AregKO mice do not produce mucous vesicles, even when asthma is induced (Figs. 3, 5, 7). Additionally, previous studies evaluating collagen fiber production by Masson trichrome staining and Sirius red staining^19^ confirm Areg as the driving factor for fibrosis and mucous cell production. However, even when asthma was induced in AregKO mice, Grocott-stained amorphous substrate increased, suggesting that increased production of amorphous substrate occurs independently of Areg-signaling activation. Since we could not define the cause of increasing amorphous substrate in his study, we think that further studies of knock-out mice are needed to define other molecules that may trigger the onset of asthma, such as IL33 and ST2^20^, using the ultrastructural analysis methods developed in this study.

As mentioned above, through ultramicro-morphological histopathological analysis using the observation method established in this study, we captured previously unidentifiable histological images, such as fibrosis, MCM, and amorphous substrate growth, leading to an understanding of signal transduction in bronchial asthma. This technique may also help analyze various diseases associated with fibrosis and mucus production and can significantly advance our understanding of each disease's pathogenesis.

## Conclusion

This method (low-vacuum SEM + Grocott staining + Os coating) enabled us to observe ultrastructural morphology in mouse bronchial tissue. This allows us to directly evaluate the remodeled histology through signal transduction and to clarify the action of Areg, a signal for bronchial asthma development. This method is expected to help elucidate the signaling pathways in various diseases associated with mucus production and fibrosis, as well as the ultrastructural morphology of pathological specimens.

## Supplementary Information

Supplementary Legends.

Supplementary Figure 1.

Supplementary Figure 2.

## Data Availability

All data generated or analysed during this study are included in this published article [and its supplementary information files].
