# Supplementary material for: The new preparation method for paraffin-embedded samples applying scanning electron microscopy revealed characteristic features in asthma-induced mice
Source: Sci Rep. 2022 May 31;12:9046. doi: 10.1038/s41598-022-12666-8 (PMC9156744; doi:10.1038/s41598-022-12666-8)
Supplement: Supplementary file 1 — Supplementary Legends. [file 41598_2022_12666_MOESM1_ESM.docx]

**Supplemental Text**

Supplementary figure 1.

Optical microscopy images of Grocott-stained lung tissue from a patient with pulmonary aspergillosis. a) Stained image at 35 minutes reaction time in methenamine silver solution. Fibers are not stained; only the fungi are stained. b) Stained image at 45 minutes reaction time. Co-staining of interstitial fibrous tissue (arrow) is observed.

Supplementary figure 2.

SEM image of Grocott-stained AregKO mouse (HDM-sensitized) bronchial tissue.

a) SEM image of bronchi and interstitium with Os coating of 2.5 nm thickness. b) SEM image of bronchi and interstitium with Nanosuit coating. c) SEM image of bronchial epithelium with Os coating of 2.5nm. d) SEM image of bronchial epithelium with Nanosuit coating.

a, b) Signal intensity of fibrous tissue is better with Os coating. c,d) Nanosuit coating specimen shows charge-up. (arrows) a,b scale bars show 200 μm. c,d scale bars show 20 μm.
