# Supplementary figures and images for: The new preparation method for paraffin-embedded samples applying scanning electron microscopy revealed characteristic features in asthma-induced mice
Source: Sci Rep. 2022 May 31;12:9046. doi: 10.1038/s41598-022-12666-8 (PMC9156744; doi:10.1038/s41598-022-12666-8)

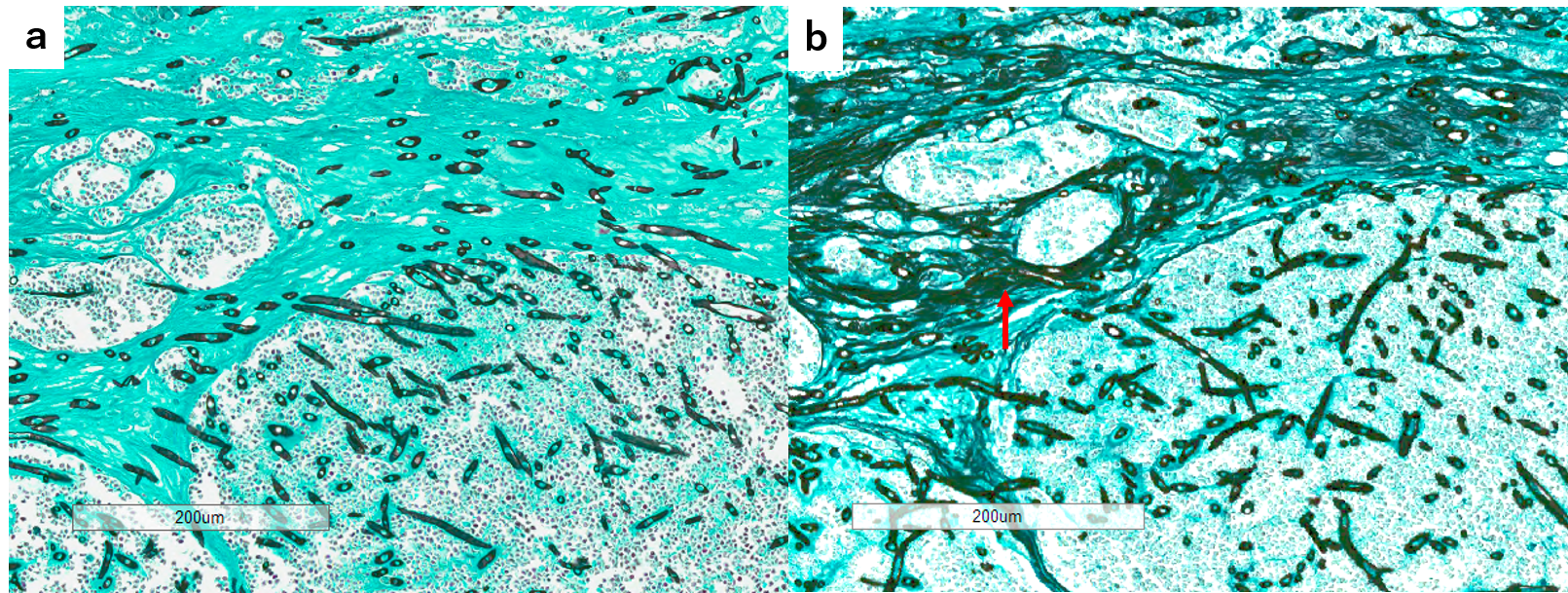

Supplement: Supplementary file 2 — Supplementary Figure 1. [file 41598_2022_12666_MOESM2_ESM.tif]

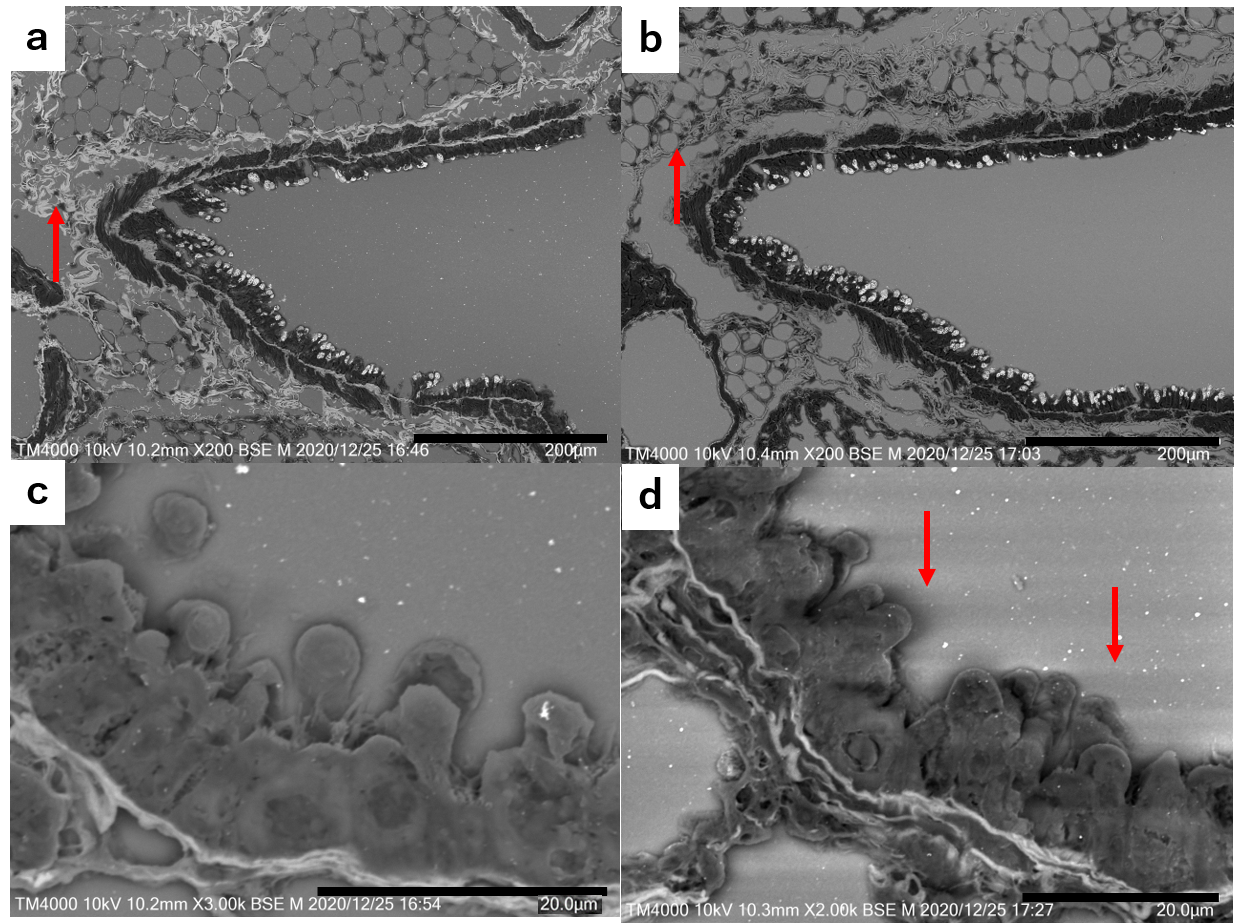

Supplement: Supplementary file 3 — Supplementary Figure 2. [file 41598_2022_12666_MOESM3_ESM.tif]
